# The Similarities and Differences Between Glomerular vs. Non-glomerular Diagnoses on Intelligence and Executive Functions in Pediatric Chronic Kidney Disease: A Brief Report

**DOI:** 10.3389/fneur.2021.787602

**Published:** 2021-12-20

**Authors:** Stephen R. Hooper, Rebecca J. Johnson, Marc Lande, Matthew Matheson, Shlomo Shinnar, Amy J. Kogon, Lyndsay Harshman, Joann Spinale, Arlene C. Gerson, Bradley A. Warady, Susan L. Furth

**Affiliations:** ^1^Department of Allied Health Sciences, School of Medicine, University of North Carolina-Chapel Hill, Chapel Hill, NC, United States; ^2^Department of Pediatrics, Children's Mercy Kansas City, School of Medicine, University of Missouri-Kansas City, Kansas City, MO, United States; ^3^Department of Pediatrics, University of Rochester Medical Center, Rochester, NY, United States; ^4^Department of Epidemiology, Johns Hopkins School of Public Health, Baltimore, MD, United States; ^5^Department of Neurology, Montefiore Medical Center, Albert Einstein College of Medicine, Bronx, NY, United States; ^6^Department of Pediatrics, Children's Hospital of Philadelphia, Philadelphia, PA, United States; ^7^Department of Pediatrics, University of Iowa Stead Family Children's Hospital, Iowa City, IA, United States; ^8^Department of Pediatrics, Rutgers Robert Wood Johnson Medical School, New Brunswick, NJ, United States; ^9^Department of Pediatrics, Johns Hopkins Medical School, Baltimore, MD, United States

**Keywords:** glomerular disease, non-glomerular disease, executive functions, CKiD study, hypertension

## Abstract

Pediatric chronic kidney disease (CKD) appears to be a heterogeneous group of conditions, but this heterogeneity has not been explored with respect to its impact on neurocognitive functioning. This study investigated the neurocognitive functioning of those with glomerular (G) vs. non-glomerular (NG) diagnoses. Data from the North American CKiD Study were employed and the current study included 1,003 children and adolescents with mild to moderate CKD. The G Group included 260 participants (median age = 14.7 years) and the NG Group included 743 individuals (median age = 9.0 years). Neurocognitive measures assessed IQ, inhibitory control, attention regulation, problem solving, working memory, and overall executive functioning. Data from all visits were included in the linear mixed model analyses. After adjusting for sociodemographic and CKD-related covariates, results indicated no differences between the diagnostic groups on measures of IQ, problem solving, working memory, and attention regulation. There was a trend for the G group to receive better parent ratings on their overall executive functions (*p* < 0.07), with a small effect size being present. Additionally, there was a significant G group X hypertension interaction (*p* < 0.003) for inhibitory control, indicating that those with both a G diagnosis and hypertension performed more poorly than the NG group with hypertension. These findings suggest that the separation of G vs. NG CKD produced minimal, but specific group differences were observed. Ongoing examination of the heterogeneity of pediatric CKD on neurocognition, perhaps at a different time point in disease progression or using a different model, appears warranted.

## Introduction

It is now known in both adult and pediatric literature that one potential health-related problem pertaining to chronic kidney disease (CKD) is the disruption of neurocognitive functioning. Hooper et al. (1–3) found that these neurocognitive difficulties are present even in children and adolescents with mild to moderate CKD, with lower performance being noted in IQ, attention regulation, and parent ratings of executive functions. Mendley et al. (4) reported specific difficulties in the area of attention regulation, particularly with longer disease duration and the presence of nephrotic proteinuria. In a comprehensive review of available pediatric findings, Chen et al. (5) also documented an array of neurocognitive difficulties, including executive dysfunction, in children and adolescents with CKD. To date, however, few studies have examined the diagnostic heterogeneity in the CKD pediatric population from a neurocognitive perspective.

Additionally, there has been emergent scientific inquiry into the mechanisms that contribute to neurocognitive difficulties in pediatric CKD that include ischemic stroke (6), lead exposure (7), mineral bone disease (8), depression (9), genetic abnormalities (10), and brain abnormalities (11). Two major suspected contributors to neurocognitive dysfunction in pediatric CKD are nephrotic proteinuria (i.e., urine protein:creatinine ≥2) (1) and hypertension (12, 13). Nephrotic proteinuria has been associated with lower neurocognitive functioning in cross-sectional studies in this population (1) and an independent correlate of CKD progression in children and adolescents with NG CKD (14); however, few studies, if any, have addressed the interaction of type of kidney disease and nephrotic proteinuria. Similarly, the presence of hypertension in pediatric CKD has been shown to be related to lower non-verbal IQ (12) and executive dysfunction, particularly set-shifting capabilities (13). While Harshman et al. (15) did not show a direct relationship between hypertension, bicarbonate, and executive functions, they did find a significant interaction between high bicarbonate and blood pressure variability with respect to parent ratings of overall executive functions. Here, higher blood pressure variability was associated with poorer parent ratings of executive functioning in the low and normal bicarbonate groups, and higher blood pressure variability was related to better parent ratings of executive function in the high bicarbonate group. As with nephrotic proteinuria, however, there have been no studies in pediatric CKD that have examined the interaction between hypertension and type of CKD.

### Glomerular vs. Non-glomerular Diagnoses

There is significant diagnostic complexity regarding the various causes for CKD in the pediatric population and a variety of strategies to attack this problem (e.g., cluster analysis, latent class modeling), but a straightforward translational approach is to begin with a clinical sorting of the various CKD diagnoses. One strategy for addressing this heterogeneity from a clinical perspective is to organize the various CKD diagnoses into glomerular vs. non-glomerular diagnoses.

Glomerular (G) diagnoses include such conditions as focal segmental glomerulosclerosis, hemolytic uremic syndrome, and systemic immunological disease such as systemic lupus erythematosus. Non-glomerular (NG) CKD diagnoses include conditions such as aplastic/hypoplastic/dysplastic kidneys, reflux nephropathy, obstructive uropathy, and congenital urologic disease. In general, children and adolescents in the NG diagnostic groups tend to be younger in terms of their age of CKD onset than the G diagnostic groups, have CKD for a greater percentage of their life given the younger age of onset, and show slower rate of CKD progression when compared to their G diagnoses counterparts. Additionally, children and adolescents with NG-CKD are more likely to be born prematurely and have a low birth weight than their peers with glomerular diagnoses. They also tend to be smaller in terms of their height and weight (16). Warady et al. (16) prospectively evaluated the progression of CKD in children and adolescents with mild to moderate CKD to either renal replacement therapy or to a 50% decline in GFR. These investigators noted that patients with NG vs. G diagnoses had differential rates of progression to the designated outcomes, and evidenced somewhat overlapping predictors of outcomes. Specifically, those with NG diagnoses had a shorter time frame to the targeted outcome with the presence of urinary protein-creatinine ratio >2 mg/mg, hypoalbuminemia, elevated blood pressure, dyslipidemia, male sex, and anemia, whereas those with G diagnoses reached the targeted outcome more quickly, in the presence of urinary protein-creatinine ratio >2 mg/mg, hypoalbuminemia, and elevated blood pressure.

While it may be tempting to predict that children and adolescent with G diagnoses may perform more poorly than children and adolescents with NG diagnoses on the neurocognitive measures, due in large part to their faster rate of disease progression, to date, few, if any, studies have examined the difference between these clinical groups on neurocognitive functioning. Several studies have examined these outcomes in targeted G and NG groups. For example, despite the overall findings provided by Hooper and colleagues (3) and Chen et al. (5) in their reviews of the CKiD findings and CKD literature to date, respectively, Hartung et al. (17) found that their small sample of children and adolescents with Autosomal Recessive Polycystic Kidney Disease (ARPKD), considered a NG disease, showed little in the way of neurocognitive impairment when compared to those with other forms of CKD. Similarly, Knight et al. (18) demonstrated that children and adolescents with lupus nephritis, considered a G disease, evidenced similar levels of neurocognitive function to their peers with other forms of glomerular CKD. In fact, Knight et al. (18) showed that these children and adolescents actually performed better than the comparison group on measures of attention regulation and problem solving.

### Current Study

The primary aim of this study was to examine the neurocognitive similarities and differences in IQ, attention regulation and related executive functions between children and adolescents with G vs. NG diagnoses. In conjunction with the significant, but subtle neurocognitive findings for the overall sample, and the relatively positive findings on the neurocognitive findings for a G condition [Lupus Nephritis (18)] and a NG condition [ARPKD (17)], we are asserting a null finding for the primary research question. It is hypothesized that children and adolescents with NG diagnoses will perform at a similar level as children and adolescents with G diagnoses across all cognitive measures. A second exploratory research question addressed the possible presence of an interaction between diagnostic grouping and two key CKD-related variables on the neurocognitive outcomes: nephrotic proteinuria and hypertension.

## Methods

### Participants

The sample included all of the available visits from participants enrolled in the NIDDK-funded CKiD Study. The CKiD Study comprises 54 clinical sites in the United States and Canada. Children and adolescents with mild to moderate CKD, ages 6 months to 16 years of age, are enrolled across sites to participate in the CKiD protocol examining issues of progression, growth, cardiovascular health, and neurocognition (19). The sample did not include children on any renal replacement therapies. All sites functioned under their university/site institutional review board with respect to recruitment and all other aspects of this study.

### Measures

Neurocognitive measures were conceptualized to assess overall intellectual abilities as well as targeted executive functions. Specific measures assessed IQ (Wechsler Abbreviated Scale of Intelligence Full Scale IQ), inhibitory control [Conners' Continuous Performance Test-II (CPT-II) Errors of Commission], attention regulation (CPT-II Variability), problem solving [Delis-Kaplan Executive Function System (D-KEFS) Tower Task Total Achievement Score], working memory (Digit Span Backwards Task from the age-appropriate Wechsler Intelligence Scale), and parent ratings of overall executive functioning (Behavior Rating Inventory of Executive Function Global Executive Composite).

In addition to subdividing the sample into G and NG diagnostic groups, additional sample description variables and targeted covariates were collected on nearly all participants at study enrollment (~96%). Sociodemographic variables included sex, race/ethnicity (for sample description only), maternal education (high school or less, some college, college or more), and chronological age at study entry. CKD-related variables included the presence of an abnormal birth history—a combined variable comprising low birth weight, prematurity, and small for gestational age, U25eGFR at study entry, (20) nephrotic proteinuria (uP/C >2), duration of CKD, age of CKD onset (i.e., ages 0–1, 2–5, 6–12, and 13 years of older), hypertension (blood pressure stage 2 or 3), (21) anemia (hemoglobin <5% threshold for chronological age, sex, race), and any history of seizures (Present/Absent).

### Data Analyses

Neurocognitive data from all available visits on participants enrolled in the CKiD Study were employed in the data analyses such that the available data on each of the measures ranged from 1,197 on the D-KEFS Total Achievement Test to 2,058 for the parent-rated BRIEF Global Executive Composite. To address the primary and secondary research questions, a series of linear mixed model regressions were conducted for all of the neurocognitive outcomes. Each linear mixed model included the G/NG diagnostic group, the targeted sociodemographic and CKD-related covariates, and interactions terms for G/NG X nephrotic proteinuria and G/NG X hypertension. For models where the interactions were not significant, the interaction terms were removed, and the simple linear mixed models were examined for the presence of differences between the CKD groups on the neurocognitive outcomes.

## Results

### Sample Characteristics

The sample included a total of 1,003 children and adolescents with mild to moderate CKD that were subdivided into those with glomerular diagnoses and non-glomerular diagnoses. As can be seen in Table 1, the glomerular group comprised about a quarter of the study participants (*n* = 260) when compared to the non-glomerular group (*n* = 743). When compared to the NG group, the G group was older (*p* < 0.0001), had significant fewer males (*p* < 0.0001), more African-Americans (*p* < 0.0001), and a similar number of participants who identified with Hispanic ethnicity (*p* = 0.55). The G group also had mothers with less education (*p* < 0.03). While the two groups were similar on the presence of an abnormal birth history (*p* = 0.28) and hypertension (*p* = 0.11), the G group demonstrated higher U25eGFR (*p* < 0.0001), a greater percentage of individuals with nephrotic proteinuria (*p* < 0.0001), shorter CKD duration (*p* < 0.0001), a generally older age of CKD onset (*p* < 0.0001), higher rates of anemia (*p* < 0.0001), and a history that included seizures (*p* < 0.0005) than the NG group.

**Table 1:** Sample description at first available visit by glomerular vs. non-glomerular diagnostic groupings.

**Characteristic**	**Median [IQR] or** ***n*** **(%)**		***p*** **-value**
	**Glomerular Dx** **(***n*** = 260)**	**Non-glomerular Dx** **(***n*** = 743)**	
Male sex	136 (52%)	495 (67%)	<0.0001
African-American race	79 (30%)	138 (19%)	<0.0001
Hispanic ethnicity	40 (15%)	103 (14%)	0.55
**Maternal education**			
High School or Less	113 (45%)	260 (36%)	
Some college	56 (22%)	204 (28%)	0.03
College or More	84 (33%)	266 (36%)	
Age, years	14.7 [11.5, 16.3]	9.0 [5.0, 13.3]	<0.0001
Abnormal birth history	69 (27%)	223 (30%)	0.28
U25eGFR, ml/min|1.73 m^2^	57.6 [42.5, 74.5]	47.3 [34.6, 61.5]	<0.0001
Nephrotic proteinuria, uP/C >2	60 (24%)	55 (8%)	<0.0001
CKD duration, years	4.0 [1.8, 8.1]	8.5 [4.7, 12.7]	<0.0001
**Age of CKD onset**			
0–1	49 (19%)	702 (95%)	
2–5	46 (18%)	11 (1%)	<0.0001
6–12	106 (42%)	18 (2%)	
13+	52 (21%)	7 (1%)	
Hypertension	54 (22%)	180 (27%)	0.11
Anemia	95 (37%)	148 (21%)	<0.0001
Seizures	44 (17%)	67 (9%)	0.0005

### Diagnostic Grouping and Neurocognitive Outcomes

As can be seen in Table 2, median scores for all of the neurocognitive variables across both groups were in the average range for chronological age. Upon initial examination of these unadjusted results, the neurocognitive scores across all of the measures did not appear to be significantly different between the two groups.

**Table 2:** Median performance at first available visit on intelligence and executive function measures by diagnostic grouping.

**Neurocognitive measures**	**Median [IQR]**	
	**Glomerular Dx** **(***n*** = 260)**	**Non-glomerular Dx** **(***n*** = 743)**
WASI-II full scale IQ	96.5 [86, 107]	98 [85, 107]
CPT errors of commission	50 [41, 57]	53 [47, 60]
CPT variability	48 [40.5, 58]	51 [43, 60]
D-KEFS tower total achievement	10 (8,11)	10 (8,11)
Wechsler digit span reverse	10 (7,11)	9.5 (7,11)
BRIEF global executive composite	52 [44, 60]	53 [45, 62]

*WASI-II Full Scale IQ has a Mean = 100, SD = 15, higher scores reflect a more intact performance. CPT and BRIEF have a Mean = 50, SD = 10, with higher scores reflecting a more impaired performance. D-KEFS and Wechsler scores have a Mean = 10, SD = 3, with higher scores reflecting a more intact performance*.

When adjusted for the targeted covariates, the linear mixed model regressions revealed that the diagnostic groupings of glomerular vs. non-glomerular disease did not seem to affect measures of IQ (*n* = 2,009, *p* = 0.85), attention variability (*n* = 1,637, *p* = 0.19), problem solving (*n* = 1,197, *p* = 0.85), or working memory (*n* = 1,277, *p* = 0.41). None of the interactions involving diagnostic grouping and hypertension and diagnostic grouping and nephrotic proteinuria were significant.

In contrast, as can be seen in Table 3, there was a trend for the diagnostic groupings to be different on the BRIEF Global Executive Composite (*n* = 2,058, *p* < 0.07). Specifically, for the BRIEF Global Executive Composite, a parent rating of overall executive capabilities, the glomerular group performed better than the non-glomerular group, with scores being ~2.35 points higher (i.e., worse ratings) and reflecting a small effect size (Cohen's d = 0.23–0.24). The interactions between diagnostic grouping, hypertension, and nephrotic proteinuria were not significant.

**Table 3:** Linear mixed model showing the model adjusted main effects for CKD diagnostic grouping on the parent-completed behavior rating inventory for executive function global executive composite (*n* = 2,058 visits).

**Predictor**	**Parameter estimate (95% CI)**	***p*** **-value**
Glomerular Dx	−2.17 (−4.52, 0.17)	0.07+
Male sex	2.39 (0.91, 3.87)	0.002^**^
Maternal education: some college	−0.30 (−2.07, 1.47)	0.74
Maternal education: college or more	−3.25 (−4.90, −1.60)	0.0001^***^
Age, per year	0.05 (−0.07, 0.16)	0.45
Abnormal birth history	1.12 (−0.43, 2.66)	0.16
U25eGFR, per 10% decline	−0.02 (−0.18, 0.13)	0.75
Percent of life with CKD, per 10%	−0.15 (−0.48, 0.18)	0.38
Nephrotic proteinuria	0.57 (−0.91, 2.06)	0.45
Hypertension	−0.09 (−1.11, 0.93)	0.86
Anemia	−0.20 (−1.25, 0.85)	0.71
Seizures	1.91 (−0.11, 3.94)	0.06+

*** *p < 0.001*;

** *p < 0.01; +p < 0.10*.

There also was a strong trend for the glomerular group to perform better than the non-glomerular group on the measure of inhibitory control, the CPT-II Errors of Commission (*n* = 1,640, *p* < 0.06); however, as can be seen in Table 4, there also was a significant interaction present between the diagnostic grouping and the presence of hypertension (*p* < 0.003). This interaction negates the main effect and indicates that those with a glomerular diagnosis and hypertension will score 4.43 points worse on this measure. To determine the effect size, the effect of having a non-glomerular disease and being hypertensive (0.94) is removed from the parameter estimate of 4.43 (4.43–0.94 = 3.49), resulting in a small effect size being present (Cohen's d = 0.34). The interaction between diagnostic grouping and nephrotic proteinuria was not significant.

**Table 4:** Linear mixed model showing the model adjusted main effects for CKD diagnostic grouping on conners continuous performance test-II errors of commission (*n* = 1,640 visits).

**Predictor**	**Parameter estimate (95% CI)**	***p*** **-value**
Glomerular Dx	−2.31 (−4.68, 0.06)	0.06+
Male sex	−2.31 (−3.76, −0.86)	0.002^**^
Maternal education: some college	−1.10 (−2.81, 0.62)	0.21
Maternal education: college or more	−3.05 (−4.67, −1.43)	0.0002^***^
Age, per year	−0.10 (−0.25, 0.05)	0.18
Abnormal birth history	−0.42 (−1.93, 1.09)	0.58
U25eGFR, per 10% decline	0.01 (−0.16, 0.18)	0.93
Percent of Life with CKD, per 10%	0.22 (−0.11, 0.55)	0.19
Nephrotic proteinuria	1.34 (−0.42, 3.1)	0.13
Hypertension	−0.94 (−2.33, 0.46)	0.19
Hypertension × Glomerular Dx	4.43 (1.54, 7.31)	0.003^**^
Anemia	−0.92 (−2.15, 0.32)	0.14
Seizures	1.71 (−0.26, 3.69)	0.09+

*** *p < 0.001*;

** *p <0.01; +p < 0.10*.

## Discussion

The primary question for this study pertained to the neurocognitive similarities and differences between children adolescents with G vs. NG diagnoses in order to address potential heterogeneity of pediatric CKD. Targeted neurocognitive outcomes included measures of intelligence, attention regulation, and related executive functions. Consistent with our null hypothesis, findings from this study revealed more similarities than differences between the G and NG groups, with IQ, attention regulation, problem solving, and verbal working memory being within the average range and unremarkable between the groups after controlling for a number of targeted sociodemographic and CKD-related factors. There was a trend for the groups to differ on a measure of inhibitory control and parent ratings of overall executive functions, with the G group performing more poorly in both instances. Additionally, there were few interactions between the groupings, nephrotic proteinuria, and hypertension on the neurocognitive outcomes. There was one significant interaction uncovered between the glomerular diagnostic group and hypertension indicating that this combination of factors contributed to poorer performance on CPT-II Errors of Commission, i.e., less inhibitory control. Consequently, while there were more similarities than differences on the neurocognitive measures, there was some sense that the G diagnostic group may be a bit more vulnerable to cognitive disruption than the NG group. Although our U25eGFR measure was not a significant predictor of neurocognitive functioning in any of the models, these findings would be consistent with the faster rate of disease progression in the glomerular diagnoses and the potentially associated neurocognitive impairment with increasing severity.

Findings revealed little in the way of neurocognitive differences between the G and NG diagnoses in children and adolescents with mild to moderate CKD, but they do suggest the potential ongoing examination of the heterogeneity of the pediatric CKD population with respect to neurocognitive functioning. In particular, the focus on the presence of hypertension and pre-hypertension should continue to be explored, particularly with respect to its impact on specific executive functions. Lande et al. (12, 13) found hypertension to not only contribute to lower non-verbal abilities, but also to set-shifting functions whereas Harshman et al. (15) found a significant interaction for high bicarbonate and blood pressure variability on parent ratings of executive functions. Further, findings from available neuroimaging studies show concerns for the vascular beds surrounding the white matter in structural imaging studies, (11, 22, 23) regional cerebral blood flow, (24) and blood flow abnormalities in both resting state (25) and working memory during fMRI (26). While these studies did not specifically examine G vs. NG diagnostic groupings, the findings do implicate the need for ongoing examination of the cardiovascular system with respect to the neurocognitive functioning in pediatric mild to moderate CKD. This also may have implications for pharmacological treatments for hypertension (Angiotensin-Converting Enzyme Inhibitors-ACES, Angiotensin Receptor Blockers-ARBS) in the pediatric CKD population with G diagnoses. While we did not find evidence of an interaction between type of kidney disease, as defined by G vs. NG diagnoses, and nephrotic proteinuria, it will be important for the impact of this factor to be tracked over time as kidney disease progresses, with perhaps a continued focus on the more rapidly progressing G diagnoses.

Additionally, it may be that the use of the dual classification of G and NG diagnoses, which is broad in nature, simply does not capture the complexities inherent in the cognitive dysfunction documented in CKD, and another type of classification strategy may be more useful in separating out those with and without neurocognitive risk. For example, Verbitsky et al. (10) reported that CKiD Study participants with genomic disorders demonstrated lower intelligence and executive dysfunction after controlling for a host of other factors. Differences also were detected with respect to the presence of anxiety and depression symptoms in those with genetic conditions and, indeed, children and adolescents with CKD and associated depression also have shown neurocognitive difficulties (9). Consequently, despite the relative lack of differences using the G vs. NG groupings in the current study, additional strategies exploring the heterogeneity of mild to moderate pediatric CKD could yield different results.

In addition, a number of other interesting possibilities could be contributing to these relationships or lack thereof. First, we wondered how repeated hospitalizations may have impacted the findings. While this is a worthwhile consideration, given the mild to moderate level of severity in our sample there actually were few repeated hospitalizations with nearly all of the sample having their health care in outpatient settings and none of them yet receiving any type of renal replacement therapy (e.g., transplant, dialysis). Furthermore, we would note that this is one of the reasons for including percent of life with CKD as a predictor in our model. A second area for ongoing exploration involves the potential relationship between age of onset and eGFR. This interaction was not included in our model due to the small correlation between variables (Rho = 0.19), so there was little need to include this in our modeling; however, it does not negate it possible influence with ongoing disease progression. A third, related possibility pertains to the relationship between age of onset and change in eGFR. This is an intriguing question as it goes to the core of whether cognitive abilities change in relationship to disease severity. For our sample, there was no correlation (*p* = 0.64) between a subject's age at CKD onset and their observed CKD progression (i.e., slope of U25eGFR over time in the study); thus, not a factor that should influence our current findings. Finally, our findings did show an independent contribution of both sex and maternal education wherein the groups differed. While these variables were automatically adjusted in the model to address the G vs. NG comparisons on the neurocognitive outcomes, the findings do raise some questions about not only how sex and lower education may impact neurocognitive outcomes. In our sample, sex produced mixed findings wherein males performed worse than females on the GEC, but better than females on the CPT-II Errors of Commission. In each instance, the effect sizes were small. In contrast, higher maternal education was related to better performance across groups on both the parent-rated GEC and the CPT-II Errors of Commission, suggesting possible protective factors of higher maternal education with respect to cognitive outcomes in pediatric CKD. Taken together, these factors provide the basis for future studies examining these key variables in the neurocognitive functioning of children and adolescents with CKD.

In summary, the groupings of G vs. NG diagnoses did not show a major differential impact on the presence of intellectual capabilities or targeted executive functions in our sample of children and adolescents with mild to moderate CKD, but those with G diagnoses did show a trend for lower overall executive functions and lower inhibitory control. Further, the glomerular X hypertension interaction also suggested that this combination of factors could lead to poor inhibitory control even after controlling for a variety of covariates. Whether the current findings will be maintained in the presence of CKD progression remains to be determined, but current knowledge supports the need for ongoing neurodevelopmental monitoring beginning with the earliest time of CKD detection. The complicated pathway of CKD toward renal replacement therapies also will demand more sophisticated types of analyses involving various interactions of key CKD-related variables with respect to the appearance of neurocognitive impairment.

## Data Availability Statement

The raw data supporting the conclusions of this article will be made available by the authors, without undue reservation.

## Ethics Statement

The studies involving human participants were reviewed and approved by University of North Carolina at Chapel and at each participating site. Written informed consent to participate in this study was provided by the participants' legal guardian/next of kin.

## Author Contributions

SH conceptualized the research, drafted the article, and worked with all authors on its completion. RJ, ML, SS, AK, LH, and JS assisted in the editing of the various drafts of the manuscript. MM contributed to the data analyses and interpretation of the findings. AG contributed to the conceptualization of the study and assisted in the editing of the various drafts of the manuscript. BW and SF conceptualized the overall CKiD design and assisted in the editing of the various drafts of the manuscript. All authors contributed to the article and approved the submitted version.

## Funding

Publication of this article was supported by The Chronic Kidney Disease in Children prospective cohort study (CKiD) with clinical coordinating centers (Principal Investigators) at Children's Mercy Hospital and the University of Missouri – Kansas City (BW) and Children's Hospital of Philadelphia (SF), central laboratory (Principal Investigator) at the Department of Pediatrics, University of Rochester Medical Center (George Schwartz), and data coordinating center (Principal Investigator) at the Johns Hopkins Bloomberg School of Public Health (Alvaro Muñoz). The CKiD is funded by the National Institute of Diabetes and Digestive and Kidney Diseases, with additional funding from the National Institute of Child Health and Human Development, and the National Heart, Lung, and Blood Institute (U01 DK066143, U01 DK066174, U01 DK082194, U01 DK066116). The CKiD website is located at http://www.statepi.jhsph.edu.

## Conflict of Interest

The authors declare that the research was conducted in the absence of any commercial or financial relationships that could be construed as a potential conflict of interest.

## Publisher's Note

All claims expressed in this article are solely those of the authors and do not necessarily represent those of their affiliated organizations, or those of the publisher, the editors and the reviewers. Any product that may be evaluated in this article, or claim that may be made by its manufacturer, is not guaranteed or endorsed by the publisher.
